# Left ventricular assist device bioinformatics identify possible hubgenes and regulatory networks involved in the myocardium of patients with left ventricular assist device

**DOI:** 10.3389/fcvm.2022.912760

**Published:** 2022-09-29

**Authors:** Maryam Ajmal, Aisha Ajmal, Maryam Rizvi, Umar Salim, Lei Huang

**Affiliations:** ^1^Faculty of Life Sciences and Medicine, Guy’s, King’s and St Thomas’ (GKT) School of Medical Education, King’s College London, London, United Kingdom; ^2^St George’s Hospital Medical School, St. George’s, University of London, London, United Kingdom; ^3^Department of Heart Center, Tianjin Third Central Hospital, Tianjin, China; ^4^Tianjin Key Laboratory of Extracorporeal Life Support for Critical Diseases, Tianjin Third Central Hospital, Tianjin, China; ^5^Artificial Cell Engineering Technology Research Center, Tianjin, China; ^6^Tianjin Institute of Hepatobiliary Disease, Tianjin, China

**Keywords:** heart failure, metabonomics, differentially expressed genes, left ventricular assist device, bioinformatic

## Abstract

**Objective:**

The aim of this study was to clarify the changes of myocardial gene expression profile after left ventricular assist device (LVAD) implantation and the related molecular biological significance.

**Methods:**

A thorough bioinformatic analysis to evaluate the changes in gene expression profile in patients pre-LVAD and post-LVAD was conducted. Four relevant gene expression datasets—GSE430, GSE974, GSE21610, and GSE52601 from Gene Expression Omnibus (GEO) database were downloaded. Analysis of GEO2R, Gene Ontology (GO), protein-protein interaction (PPI) were used to determine differentially expressed genes (DEGs) and their function, respectively.

**Results:**

A total of 37 DEGs were identified, including 26 down-regulated and 11 up-regulated genes. The molecular function of DEGs were enriched in “cytokine activity,” “neurotransmitter binding,” “receptor ligand activity.” The gene set enrichment analysis (GSEA) revealed an overall marked increase of neutrophil degranulation signaling, closely correlated with the G protein coupled receptor (GPCR)—ligand binding process after LVAD assistance. 16 hubgenes in these DEGs were further selected and the biological process involved is mainly related to positive regulation of leukocyte chemotaxis mediated by chemokines.

**Conclusion:**

Inflammatory signaling pathway is crucial for the pathophysiology after LVAD implantation. Chemokines mediate cardiac inflammatory response and tissue remodeling after LVAD implantation through GPCR—ligand binding.

## Background and aim

Heart failure with reduced ejection fraction (HFrEF) remains one of the leading causes of morbidity and mortality worldwide, and approximately 5% of heart failure patients face end-stage disease, refractory to medical therapy (1). Currently, there is no bottleneck solution for end-stage HF, except through heart transplantation. However, factors such as unsuitable match and advanced patient age make the number of heart transplants performed globally a limited option against demand. More recently, however, the implementation of mechanical circulatory support (MCS) such as left ventricular assist device (LVAD), have transformed the outlook toward prognosis for such HF patients (2).

LVAD support can improve cardiac function, as the alternative pathway created by the cannula overcomes the effects of the stiffened ventricular myocardium and the subsequent inability to sufficiently eject blood to the rest of the body. Reduced ventricular strain and increased ventricular outflow translate with the myocardium undergoing structural and molecular changes *via* a process known as “reverse remodeling,” which describes the transposing of the adverse pathophysiological remodeling seen in the myocardium during the development of heart failure with preserved ejection fraction.

Emerging studies have asserted that despite the reverse remodeling impacts seen during LVAD support, this is not a pervasive process. Incomplete recovery of gene expression involved with metabolism remain examples of myocardial and/or ventricular properties that do not comply with the reverse remodeling and do not revert toward normal during LVAD assistance (3). Furthermore, there remains little understanding and explanation as to why substantial and sustained cardiac recovery during LVAD support is demonstrated in only some patients (4), reinforcing the notion that although the expressions of specific genes of interest can normalize following LVAD assistance, the expressions of many genes remain abnormal. Therefore, the study aims to evaluate the changes in gene expression profile in myocardial tissue pre-LVAD and post-LVAD, identify potential hubgenes and the associated signaling pathways involved *via* comprehensive bioinformatic analysis. Although our study is not the first to use bioinformatics analysis to investigate the effects of LVAD on HFrEF patients (5), we offer an extension to the field by using a larger Gene Expression Omnibus (GEO) dataset, with a larger sample size, and a range of targets for prospective studies, to hopefully investigate impediments for the molecular mechanisms that are involved in post-LVAD pathophysiology.

## Materials and methods

### Transcriptome data

The mRNA expression of advanced heart failure who had LVAD treatment was searched in the GEO database.^1^ An advanced search was carried out for the selection of appropriate datasets by introducing the search phrases “LVAD/LVAD” and “Homo sapiens” [porgn: txid9606]. A list of all end-stage heart failure datasets with LVAD datasets with human mRNA expression levels was collected. Only those datasets that met the following inclusion and exclusion criteria were considered for further analysis: (i) datasets comprising whole cardiac tissue mRNA expression levels of HFrEF patients who underwent LVAD; (ii) datasets reporting mRNA expression levels of each HFrEF patient pre- and post-LVAD therapy, regardless of whether a healthy control group was included. Exclusion criteria: datasets having information about the expression of other types of biological tissue samples (e.g., plasma) but not myocardial tissue.

As a result, the GEO database yielded four gene expression profiles: GSE430, GSE974, GSE21610, and GSE52601. 7 patients with congestive heart failure whose myocardial tissue was acquired pre and post LVAD treatment for heart transplantation were included in the GSE430 transcriptome data (7 paired samples). Pre and post mechanical unloading, GSE21610 included 8 non-failing control hearts and 30 patients with end-stage heart failure (30 paired samples). GSE974 included five cases of ischemic cardiomyopathy with evidence of coronary artery disease (CAD), eight cases of dilated cardiomyopathy with no evidence of CAD, and six cases of acute myocardial infarction (MI) within 10 days of the implant. 9 non-failing myocardium samples (5 adults and 4 fetuses) and 8 paired myocardial samples from progressive heart failure (4 dilated cardiomyopathy and 4 ischemic cardiomyopathies) were included in GSE52601.

### Data processing and differently expressed gene identification

To identify genes that are differently expressed in cardiac tissue between pre and post-LVAD treatment, the raw transcriptome data from the four datasets acquired from the GEO database were processed using the web tool GEO2R.^2^ The analysis’ exact threshold value was based on our previous research (6). To show the overlap of DEGs between the three discovery datasets, Venn diagrams were produced using Venn Diagrams software.^3^ DEGs from at least two dataset were chosen for further investigation.

### Functional and pathway enrichment analysis

Using the Database for Annotation, Visualization, and Integrated Discovery (DAVID, version 6.8)^4^ software, upregulated and downregulated DEGs were subjected to Gene Ontology (GO) and Kyoto Encyclopedia of Genes and Genomes (KEGG) pathway analysis. It was determined that *P* < 0.05 was statistically significant. The gene set enrichment analysis (GSEA) program and the GSEA pre-ranked function were used to execute GSEA on the Molecular Signature Database (v6.1). FDR < 0.25 and p.adjust < 0.05 are the significant enrichment thresholds.

### Protein-protein interaction network construction and module analysis

The protein-protein interaction (PPI) network was predicted using the internet database Search Tool for the Retrieval of Interacting Genes (STRING, version 10.0).^5^ The STRING database was used to build a PPI network of DEGs in this investigation, and interaction with a combined score > 0.4 was considered statistically significant. The molecular interaction networks were visualized using Cytoscape (version 3.8.0), with Molecular Complex Detection (MCODE) scores > 5, degree cut-off = 2, node score cut-off = 0.2, max depth = 100, and k-score = 2 set as the cut-off parameters.

## Results

### Selection of gene expression omnibus datasets

We performed a systematic review of GEO datasets concerning HFrEF patients with LVAD. The search criteria are as follows: “left ventricular assisting device/LVAD” as the keywords, “*homo sapiens”* as the research species, “GEO Datasets” as the data source (search time up to June 1, 2021). A total of four datasets GSE430, GSE974, GSE21610, and GSE52601 were selected.

### Identification of differentially expressed genes

The differential analysis performed by GEO2R on the four gene expression datasets allowed the identification of a list of dysregulated genes in HFrEF patients with LVAD compared to normal healthy controls. First, a principal component analysis (PCA) model for the DEGs of each dataset was established for HFrEF patients before and after LVAD implantation. The score plots of their first two principal components are shown in Supplementary Figure 1, demonstrating a distinct separation between them on the score plots, suggesting that the transcriptome of cardiomyocytes has indeed changed significantly after LVAD treatment, and the DEGs have a robust ability to distinguish the paired samples before and after the intervention.

**FIGURE 1:**
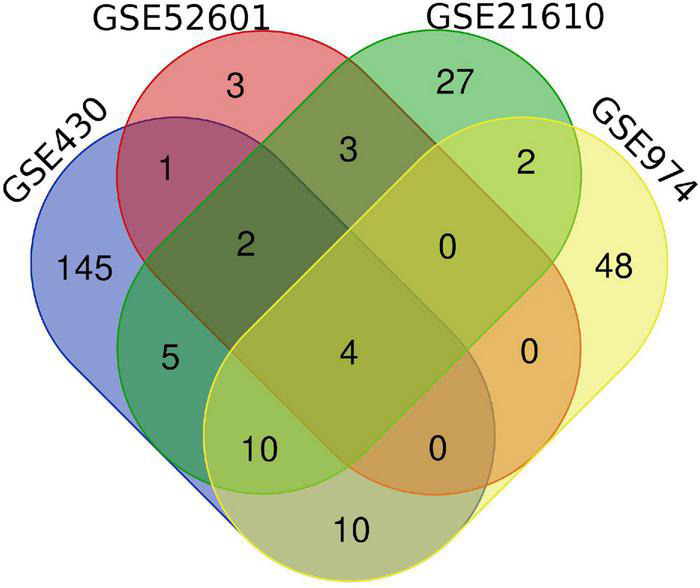
Venn diagram of differentially expressed genes from four transcriptome datasets.

**FIGURE 2:**
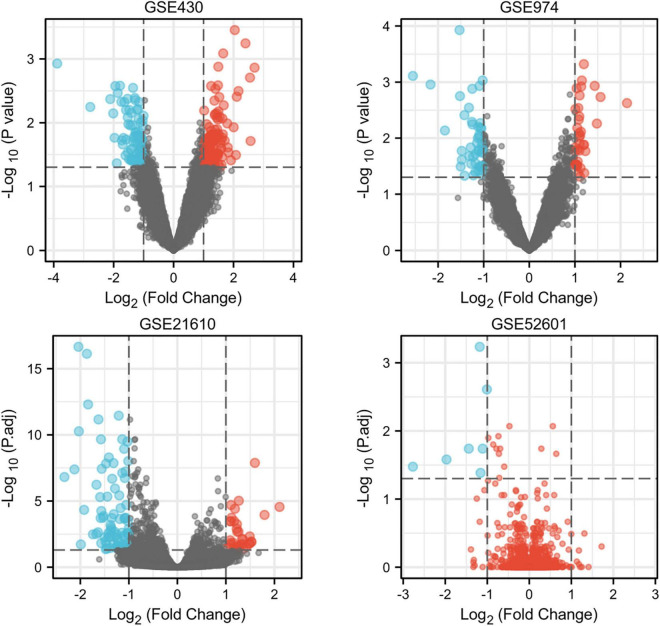
The volcano plots of differentially expressed genes in four datasets. Red indicates genes with higher levels of expression, blue indicates genes with lower levels of expression, and gray indicates genes with no differential expression based on the criteria of P 0.05 and |logFC| 1.0, respectively. P.adj, adjusted *P* value.

**FIGURE 3:**
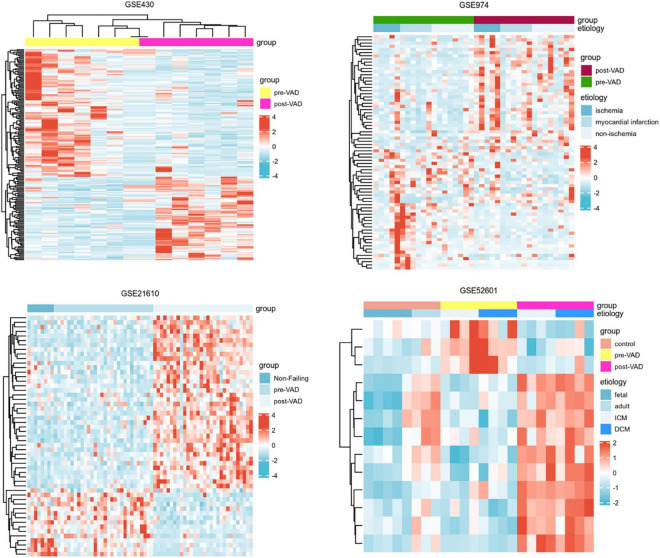
Heatmaps of differentially expressed genes in the four transcriptome datasets showed the hierarchical clustering characteristics of gene transcription changes in a cluster analysis of different groups. VAD, ventricular assist device; ICM, ischemic cardiomyopathy; DCM, dilated cardiomyopathy.

Next, a total of 37 common DEGs were obtained (Figure 1), of which 26 were up-regulated and 11 were down-regulated (Table 1). The volcanic maps more intuitively showed the distribution of up-and down-regulated DEGs in HFrEF patients pre-LVAD point compared to the post-LVAD group (Figure 2). The heatmap of DEGs in these four microarrays showed hierarchical clustering of altered transcription in various groups (Figure 3) that may facilitate identification of the function of unknown transcripts or the unknown function of known transcripts by collecting similar expression patterns. The results from the four datasets consistently showed significant changes in myocardial transcriptome before and after LVAD treatment in patients with end-stage heart failure. Interestingly, the clinical and microarray results from GSE52601 showed that the transcriptome of heart failure before LVAD treatment had a similar transcriptome profile as the one from the healthy fetus. Although LVAD did not significantly improve the value of left ventricular ejection fraction, it seems to shift the transcriptome profile of failed myocardium to that of healthy adults, in contrast to the fetal heart seen before the LVAD intervention.

**TABLE 1:** 37 DEGs were identified from GSE430, GSE974, GSE21610, and GSE52601.

DEGs	Gene symbol
Upregulated (25)	***GADD45G, DDIT4, FKBP5*,** *ZNF189, RGCC, BTG2, ZBTB16*, *AREG, MT1M, CYP4B1, FOXO3B///FOXO3, USP53, FOXO3, KLF9, RASAL2, CPM, PDE4DIP, CNN1, KCNK1, PNMT, PDK4, BAIAP2, MT1X, ARRDC2, TSC22D3,RASD1*
Downregulated (11)	***CCL2***, *G0S2, RGS4, PDE4B, CD14, CX3CR1, SELE, HS3ST3A1, ARRB1, PLXNC1, PTGFR*

The 4 genes in bold italics are the common DEGs to the four datasets. DEGs, differentially expressed genes.

**FIGURE 4:**
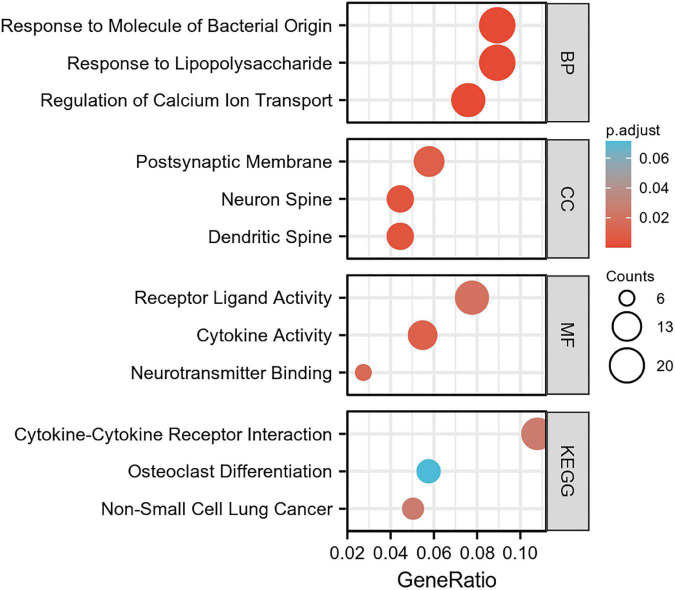
The GO enrichment analysis and KEGG pathway of differentially expressed genes in HF patients before and after LVAD implantation. The color depth of nodes refers to the corrected *P*-value of ontologies. The size of nodes refers to the numbers of genes that are involved in the ontologies. *P* < 0.01 was considered statistically significant.

**FIGURE 5:**
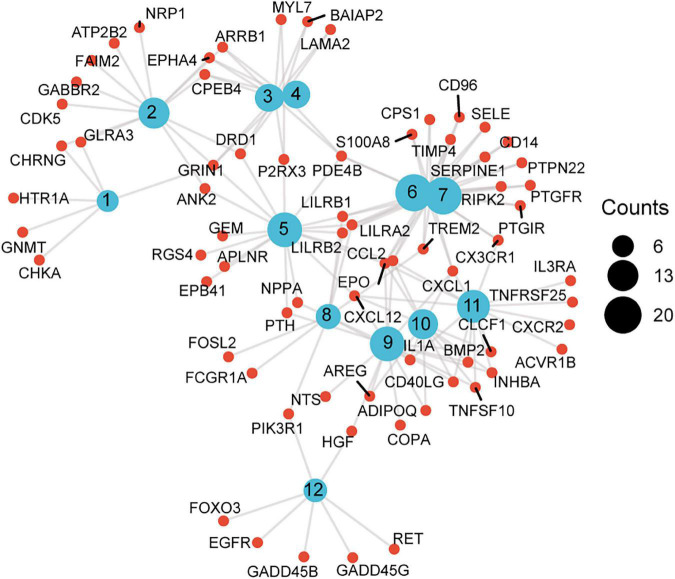
A scattergram composed of GO description and associated genes. Blue nodes represent GO terms, red nodes represent differentially expressed genes (DEG), and the lines indicate that a DEG has a corresponding GO annotation. The node size is consistent with the number of counts. 1. Neurotransmitter binding, 2. postsynaptic membrane, 3. neuron spine, 4. dendritic spine, 5. regulation of calcium ion transport, 6. response to molecule of bacterial origin, 7. response to lipopolysaccharide, 8. osteoclast differentiation, 9. receptor ligand activity, 10. cytokine activity, 11. cytokine-cytokine receptor interaction, 12. non-small cell lung cancer.

To elucidate the changes in differential gene expression profiles and their functions in HFrEF pre- vs. post-LVAD, the pooled DEGs from the four datasets were annotated by GO analysis. According to the GO analysis results from the DAVID database, the changes in biological processes of DEGs were significantly enriched in “response to lipopolysaccharide,” “response to molecule of bacterial origin,” “regulation of calcium ion transport”; the changes in molecular function were enriched in “cytokine activity,” “neurotransmitter binding,” “receptor ligand activity”; and the changes in cell component of DEGs were enriched in “dendritic spine,” “neuron spine,” “postsynaptic membrane.” KEGG pathway analysis revealed that the pathways enriched by dysregulated DEGs included “cytokine-cytokine receptor interaction,” “non-small cell lung cancer” and “osteoclast differentiation” (Figure 4 and Table 2). The GO description and genes involved were organized as a scattergram (Figure 5). GSEA revealed an overall marked increase of neutrophil degranulation signaling, closely correlated with the GPCR ligand binding process. The details of the GSEA analysis of the four datasets was shown in Table 3.

**TABLE 2:** GO and KEGG pathway enrichment analysis of DEGs (Top 5 terms were listed).

Term	Description	Count	*P*-value	*q*-value
**Biological process**				
GO:0032496	Response to lipopolysaccharide	20	3.32e-09	9.11e-06
GO:0002237	Response to molecule of bacterial origin	20	6.41e-09	9.11e-06
GO:0051924	Regulation of calcium ion transport	17	1.21e-08	1.15e-05
GO:0006816	Calcium ion transport	21	6.72e-08	4.78e-05
GO:0070838	Divalent metal ion transport	21	3.99e-07	2.26e-04
**Cell components**				
GO:0043197	Dendritic spine	10	2.52e-05	0.004
GO:0044309	Neuron spine	10	2.79e-05	0.004
GO:0045211	Postsynaptic membrane	13	9.20e-05	0.008
GO:0097060	Synaptic membrane	15	1.40e-04	0.009
**Molecular function**				
GO:0005125	Cytokine activity	12	1.98e-05	0.010
GO:0042165	Neurotransmitter binding	6	5.86e-05	0.014
GO:0048018	Receptor ligand activity	17	1.09e-04	0.018
GO:0005516	Calmodulin binding	10	2.00e-04	0.020
GO:0004955	Prostaglandin receptor activity	3	2.10e-04	0.020
**KEGG pathway**				
hsa04060	Cytokine-cytokine receptor interaction	15	1.58e-04	0.023
hsa05223	Non-small cell lung cancer	7	2.23e-04	0.023
hsa04380	Osteoclast differentiation	8	0.002	0.064
hsa05144	Malaria	5	0.002	0.064
hsa04933	AGE-RAGE signaling pathway in diabetic complications	7	0.002	0.064

DEGs, differentially expressed genes; FDR, false discovery rate; GO, Gene ontology; KEGG, Kyoto Encyclopedia of Genes and Genomes.

**TABLE 3:** The results of GSEA analysis in GSE430, GSE974, GSE21610, and GSE52601.

Geneset enrichment description	ES	NES	p.adjust	FDR
**GSE974**				
Neutrophil degranulation	0.469	1.797	0.141	0.127
Toll like receptor cascades	0.455	1.636	0.141	0.127
**GSE21610**				
G alpha I signaling events	0.395	1.352	0.118	0.109
Neuroactive ligand receptor interaction	0.43	1.454	0.118	0.109
**GSE52601**				
Signaling by interleukins	0.357	1.366	0.141	0.127
Signaling by receptor tyrosine kinases	0.332	1.278	0.191	0.173
**GSE430**				
Neutrophil degranulation	0.424	1.441	0.078	0.073
GPCR ligand binding	0.49	1.659	0.078	0.073

FDR, false discovery rate; GPCR, G protein-coupled receptors.

### Protein-protein interaction network analysis

**FIGURE 6:**
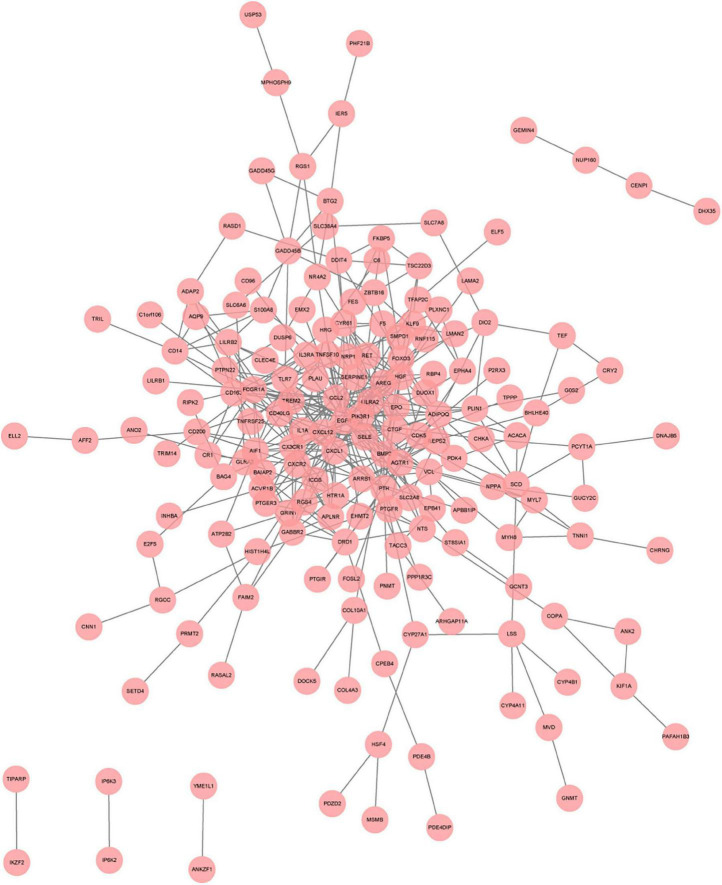
A network panorama of protein-protein interaction composed of all differentially expressed genes of heart failure patients before and after LVAD implantation. Lines between nodes represent interactions between genes.

**FIGURE 7:**
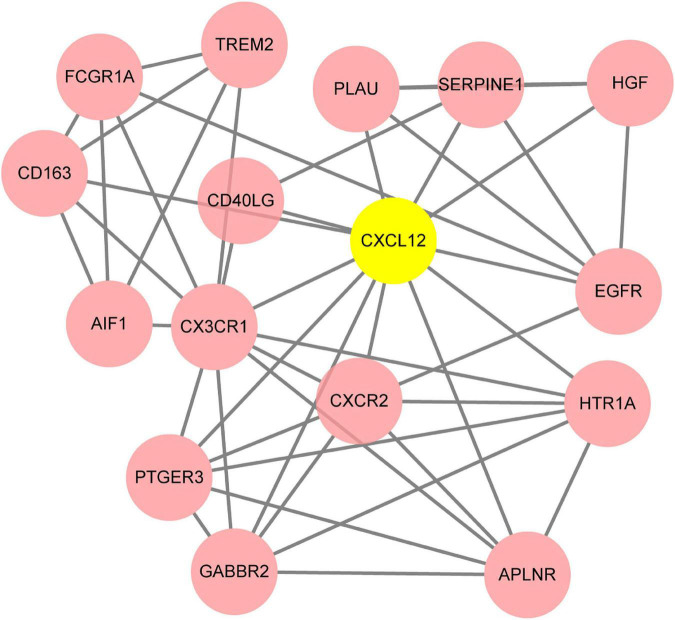
The key module identified based on MCODE (Molecular Complex Detection) plug-in analysis. The gene of CXCL12 highlighted in yellow is the seed gene of the whole module.

To systematically analyze biologic functions of obtained DEGs between two groups, the PPI network of DEGs was constructed using Cytoscape with protein interaction information. With a PPI score > 0.4, a PPI network with 231 nodes and 449 edges was constructed (Figure 6). Nine modules consisting of 43 hubgenes were obtained from a PPI network of DEGs using MCODE. A total of 16 genes were identified as hub genes with degrees ≥ 10 (Figure 7). The names, abbreviations, and functions for these hub genes are illustrated in Table 4. The molecular function analysis of the hubgenes is mainly related to signal transduction activity mediated by chemokines (CXCL12—CX3CR1/CXCR2) *via* their cell transmembrane G protein coupled receptors (GPCRs). The biological process analysis of the hubgenes is mainly related to “regulation of defense response to proinflammatory stimulus” “positive regulation of leukocyte chemotaxis, cell proliferation and apoptosis” (Supplementary Figure 2).

**TABLE 4:** Functional roles of 16 hub genes with degree ≥ 10.

Gene symbol	Full name	Function
EGFR	Epidermal growth factor receptor	Receptor tyrosine kinase binding ligands of the EGF family
APLNR	Apelin receptor	Regulation of blood vessel formation, heart contractility, and heart failure by acting as a receptor for APLN hormone
AIF1	Allograft inflammatory factor 1	Plays a role in RAC signaling, in phagocytosis, and in macrophage activation and function. Promotes the proliferation of vascular smooth muscle cells and of T- lymphocytes. Enhances lymphocyte migration
GABBR2	Gamma-aminobutyric acid type B receptor subunit 2	Mediates coupling to G proteins. Ligand binding and modulates the activity of down-stream effectors, such as adenylate cyclase.
CX3CR1	CX3C chemokine receptor 1	Binds to CX3CL1 and mediates both its adhesive and migratory functions
HTR1A	5-hydroxytryptamine receptor 1A	Ligand binding that triggers signaling *via* G proteins and modulates the activity of down-stream effectors, such as adenylate cyclase.
PLAU	Urokinase-type plasminogen activator	Specifically cleaves the zymogen plasminogen to form the active enzyme plasmin
CXCR2	C-X-C chemokine receptor type 2	Receptor for interleukin-8 which is a powerful neutrophil chemotactic factor.
CD163	Scavenger receptor cysteine-rich type 1 protein M130	Acute phase-regulated receptor involved in clearance and endocytosis of hemoglobin/haptoglobin complexes by macrophages, protect tissues from free hemoglobin-mediated oxidative damage.
FCGR1A	High affinity immunoglobulin gamma Fc receptor I	High affinity receptor for the Fc region of immunoglobulins gamma. Functions in both innate and adaptive immune responses
PTGER3	Prostaglandin E2 receptor EP3 subtype	Inhibition of adenylate cyclase mediated by G-I proteins, and to an elevation of intracellular calcium.
HGF	Hepatocyte growth factor	Potent mitogen for mature parenchymal hepatocyte cells, a growth factor for a broad spectrum of tissues and cell types.
CXCL12	Stromal cell-derived factor 1	Chemoattractant active on T-lymphocytes, monocytes, but not neutrophils. Inducing a rapid and transient rise in the level of intracellular calcium ions and chemotaxis
SERPINE1	Plasminogen activator inhibitor 1	Function as a major control point in the regulation of fibrinolysis
CD40LG	CD40 ligand	Co-stimulates T-cell proliferation and cytokine production

## Discussion

In this study, a thorough bioinformatic analysis to evaluate the changes in gene expression profile in patients pre-LVAD and post-LVAD from four relevant gene expression datasets was conducted. In summary, the GSEA of the DEGs revealed an overall increase in neutrophil degranulation signaling after LVAD assistance. Furthermore, 16 hubgenes in these DEGs were mainly related to positive regulation of leukocyte chemotaxis mediated by chemokines, mediating deleterious pathophysiological changes in the myocardium post-LVAD and delved deeper into these processes.

We focus on the biological significance and potential therapeutic value of these pivotal genes in the pathophysiology process of heart failure and post-LVAD myocardial remodeling.

### PTGER3

The *PTGER3* gene represents the prostaglandin E2 (PGE2) receptor EP3 subtype gene. It is usually expressed in response to rising intracellular calcium. Maintenance of Ca^2+^ homeostasis is vital to regulating the normal contractile strength of the heart (7). Altering the Ca^2+^ cycle (often *via* downregulated gene expression of *SERCA2a*) appears to correlate with contractile dysfunction.

Whether the PGE2 receptor contributes to contractile dysfunction remains debated, with many authors discouraging the role of PGE_2_ in regulating catecholamine-induced inotropy (8), and with Klein et al. (9) observing that PGE_2_ augmented peak shortening in adult rat cardiomyocytes remained independent of alteration in calcium.

Recently it was discovered that PGE_2_ can directly influence cardiac contractility through both positive and negative inotropic effects, as mediated by its EP3 receptor (10), thereby altering the available concentration of Ca^2+^ for the excitation–contraction coupling.

The deleterious effects of altered Ca^2+^ homeostasis in contributing to post-LVAD cardiac remodeling, as influenced by the PGE_2_ EP3 receptor, can be elaborated upon by the subsequent dephosphorylation of phospholamban. Phospholamban is a reversible regulator of SERCA2a activity, thereby regulating cardiac contractility. Usually, phospholamban is phosphorylated, and physically dissociates from SERCA2a, whereby releasing large amounts of Ca^2+^ from the sarcoplasmic reticulum. Under PTGER3 expression, however, phospholamban transforms to its dephosphorylated form. Consequently, phospholamban modulates Ca^2+^ load, contractility, and relaxation, leading to the pathophysiological events underpinning HF development.

Downregulation of the Ca^2+^ cycling is an unfavorable consequence of post-LVAD remodeling (11, 12), but the explanations of this remain unclear. Our study bridges the link between such adverse Ca^2+^ cycling that is commonly reported post-LVAD, as being accounted for by the increased expression of the PTGER gene post-LVAD. Therapeutically, considering the positive lucistropic and vasodilator effects seen in healthy volunteers following administration of a selective EP4 agonist (13), administering EP4 alongside LVAD placement may serve as a viable therapeutic route in aiding to overcome the adverse post-LVAD pathophysiology—importantly, when considering the increased ejection fraction and decreased end systolic pressure seen in anesthetized dogs (14) following acute infusion of an EP4 agonist.

### Epidermal growth factor receptor

In the heart, a GPCR Kinase β-arrestin signaling appears to enhance cardiomyocyte function and survival. This antagonizes the conventional pathway of coupled second messenger molecule activation by G protein activation resulting in Canonical GPCR signaling. G protein–mediated signaling enhances cardiomyocyte function acutely, albeit at the expense of long-term function and survival of the cardiomyocytes. LVAD support has been shown to inhibit chronic GPCR activation (15).

GPCRs are activated by cardiovascular mechanical load. Strain-induced activation of the angiotensin II type 1 receptor (AT_1_R) results in β-arrestin2 activation, which couples the AT_1_R to transactivate the EGFR, a tyrosine kinase receptor. Studies (16) have shown the interaction between β-arrestins and EGFR decreases due to LVAD intervention, resulting in a relatively reduced left ventricular end-diastolic volume and diastolic myocardial strain. However, we found the EGFR hubgene to be elevated post-LVAD. This suggests the duration and amount of mechanical load may influence the activation of GPCRs and hence, the expression of EGFR, whereby influencing the optimal point for the duration and extent of LVAD support.

### CD40LG, CXCL12, AIF1, CXCR2, FCGR1A and other pro-inflammatory genes

Our results from the KEGG analysis highlighted “cytokine-signaling” as a key factor causing post-LVAD pathophysiology. We identified hubgenes *CD40LG*, *AIF1*, and *CXCL12* and *CXCR2*, amongst others, which involve cytokine-signaling and T-cell recruitment. The *CD40L* hubgene is responsible for co-stimulating T-cell proliferation and cytokine production. The *CXCL12* gene encodes stromal cell-derived factor 1α (SDF-1), also known as C-X-C motif chemokine 12 (CXCL12) and can be induced by pro-inflammatory stimuli to activate leukocytes. The *CXCR2* hubgene, encoding for the CXCR2 receptor found on neutrophils, functions as a receptor for interleukin 8 (IL-8). IL-8 binds to the receptor with great affinity, facilitating the clustering of neutrophils to inflammatory sites. The FCGR1A gene encodes for the high affinity Fc-gamma receptor, for IgG, which plays a significant role in both innate and adaptive immune responses. These genes may contribute to the pathophysiology of HF, as evidently, the derangement of chemokine expression gears the progression of end-stage HF (17, 18).

Interestingly, SDF-1 has directly been associated with HF and all-cause mortality risk (19). Recent studies have found a link between the genomic locus of the *CXCL12* gene and a risk for CAD (19). The Bruneck study demonstrates plasma SDF-1 levels as being inversely proportional to circulating endothelial progenitor cell numbers (20), reinforcing the role of CXCL12 hubgene in the post-LVAD pathophysiology. As such, both can be plausible developmental benchmarks for post-LVAD, as both CAD and endothelial dysfunction are changes seen in the myocardium post-LVAD. Further studies have offered extensions into understanding the role of the SDF-1 and MI, with Uematsu et al. reporting the adverse left ventricular remodeling and progressive dysfunction seen in acute MI survivors being linked with the production of SDF-1 in the infarcted myocardium in the chronic phase of MI (21).

Another key discovery is the association between the CXCL12 hubgene and the development of atherosclerosis (22). The pro-inflammatory molecule, AIF1, is also associated with the development of atherosclerosis and is expressed primarily in the monocyte/macrophage (MP) lineage, and is a positive regulator of the NF-κB pathway (23). The loss of AIF1 may decrease NF-κB-dependent inflammatory gene expression and hence, may decrease plaque size (22). The resultant hypoxic situation further drives the increased expression of CXCL12 and CXCR4. A study involving cardiomyocyte-specific CXCL12-overexpressing transgenic rats revealed impaired cardiac function post-MI, accompanied by enhanced fibrosis (24).

Albeit numerous studies contend to the deleterious role of such CXCL12/CXCR4 signaling following MI, the standpoint remains unclear, especially when considering the survival effects enforced on resident cardiomyocytes and the subsequent recruitment of protective cells (25). Furthermore, myocardial infarct size following an ischemic episode has been reduced by the injection of CXCL12. The recruitment of progenitor cells (26) and the subsequent increased neo-angiogenesis have been postulated as the elucidative events.

In concurrence, a study shows that AMD3100, an antagonist for CXCR4, improves cardiac function and enhances neovascularization and progenitor cell assemblage (27). However, results also contended that a reduced recruitment of progenitor cells was seen after long term AMD3100 administration, and was associated with a poor cardiac outcome (28). Collectively, results imply that the protective effects of CXCR4 remain time-dependent, with shorter term exposure leading to detrimental outcomes. The confounding results necessitate a more profound understanding of the overall role of the CXCL12/CXCR4 in regulating cardiac signaling after MI and indicates the need for further studies in this field.

Overall, the role of cytokine-signaling in the post-LVAD pathophysiology is evidently associated with the events that determine adverse ventricular remodeling, as seen by Grosman-Rimon et al.’s report (29), who demonstrate recipients with continuous-flow LVADs as demonstrating a higher level of inflammatory biomarkers and specific expression changes in the chemokine signaling pathway. Contrastingly, Nayak et al. (30) found increased mortality was associated with the downregulation of chemokine signaling, and these effects were translated by the progression toward right heart failure—one of the fatal complications of LVAD assistance. Disparities maybe due to the different types of devices, or the lengths of time the LVAD assistance remained in place, but the validation of this requires further study and investigation.

### CX3C chemokine receptor 1

CX3C chemokine receptor 1 (CX3CR1) is expressed on natural killer cells, monocytes, smooth muscle cells and T cells, and mediates migration, adhesion, and proliferation. CX3CR is involved in chemokine binding. Nayak et al. found that when chemokine signaling was downregulated, it increased mortality and facilitated the fatal adverse effects after LVAD implantation, particularly, the process of right heart failure (30).

Additionally, Richter et al. assessed the plasma levels of the fractalkine ligand (CX3CL1) for the receptor, CX3CR1, in 349 patients with advanced systolic HF and found that CX3CL1 was a significant predictor of all-cause mortality with a hazard ratio for the third compared to the first tertile of 2.78 (31).

Thus, targeting the binding of FKN to its receptor, CX3CR1, may be valuable in overcoming the post-LVAD contribution to cardiac remodeling.

### Hepatocyte growth factor

The hepatocyte growth factor (HGF) gene has been associated with cardioprotective effects (32), but a study researching the effects of Insulin-like growth factor 1 (IGF-1) and HGF-overexpression in pig mesenchymal stem cells from adipose tissue (paMSC) (33) demonstrated neovascularization and fibrosis, suggesting both beneficial and detrimental effects of these genes, and overall do not improve cardiac regeneration. It is unclear which gene has which effect or if both work synergistically to display the observed trend. Other studies have demonstrated the involvement of myocardial IGF-1 mRNA in promoting cardiac recovery. IGF-I may act to limit the atrophy and apoptosis that is seen during adverse remodeling post-LVAD, and to therefore promote repair and regeneration (34). In advanced HF, the HGF gene is a convincing predictor of mortality as it exhibits unfavorable effects.

Circulating HGF is associated with the incidence of stroke, demonstrating HGF as a marker of endothelial damage (35). Normal functioning endothelium provides several therapeutic effects, such as anti-proliferative and anti-inflammatory actions—the disturbance of which can be implicated in the pathophysiology of HF. Dysfunctional endothelium can therefore be responsible for exacerbating the already existing vasoconstriction in patients, increasing afterload due to systemic and pulmonary vascular constriction, and compounding myocardial damage further.

Increased understanding of normal endothelial functioning can help limit post-LVAD pathophysiology. Recent studies asserted the increasing involvement of allopurinol as a xanthine oxidase (XO) inhibitor, in the maintenance of normal endothelial relaxation (36). Allopurinol inhibits the binding of circulating XO to the endothelial surface, where reactive oxygen species are produced, which may enhance endothelial function in CHF patients. When coupled with the improved endothelial function seen in a study of 197 patients with CHF with LV systolic dysfunction following administration of 300 mg allopurinol for 1 week to 3 months (37), provides convincing evidence to incorporate allopurinol as an adjunctive measure alongside LVAD. Furthermore, allopurinol demonstrates favorable effects on the regression of left ventricular hypertrophy, ventricular remodeling, myocardial energy metabolism, myocardial oxygen consumption, and clinical status in the setting of HF. Adding allopurinol in the mediation of the post-LVAD pathophysiology seems viable, but the lack of existing data necessitates further research into factors mediating this protective effect given the post-LVAD setting.

## Conclusion

In the present study, we have *identified* 37 DEGs, of which 26 were downregulated and 11 were upregulated. We were then able to identify 16 hub genes that are involved in the adverse pathological remodeling seen post-LVAD. We explored the deleterious pathophysiological process that could be occurring in the myocardium, under the influence of the hubgenes. Our findings may be useful in designing investigations that combine LVAD unloading and pharmacological therapy as a bridge to cardiac recovery of the failing heart.

## Data availability statement

Publicly available datasets were analyzed in this study. This data can be found here: https://www.ncbi.nlm.nih.gov/geo/query/acc.cgi?acc=GSE430; https://www.ncbi.nlm.nih.gov/geo/query/acc.cgi?acc=GSE974; https://www.ncbi.nlm.nih.gov/geo/query/acc.cgi?acc=GSE21610; https://www.ncbi.nlm.nih.gov/geo/query/acc.cgi?acc=GSE52601.

## Ethics statement

Ethical review and approval was not required for this study in accordance with the local legislation and institutional requirements.

## Author contributions

MA and LH contributed to research design, performance, and data analysis. MA, AA, MR, and LH contributed to manuscript writing. MA, AA, LH, MR, and US involved in the critical evaluation and intellectual contribution to the manuscript. All authors have read and approved the manuscript.

## Footnotes

1
http://www.ncbi.nlm.nih.gov/geo

2
http://www.ncbi.nlm.nih.gov/geo/geo2r

3
http://bioinformatics.psb.ugent.be/webtools/Venn/

4
https://david.ncifcrf.gov/home.jsp

5
http://string-db.org

## Supplementary material

Supplementary Figure 1 Principal component analysis (PCA) score plots of the differentially expressed genes in each dataset, indicating that the sum of the first two principal components (PC1 and PC2) reflects the difference of more than half of the data (46.2–82%).

Supplementary Figure 2 The biological process analysis of the hubgenes in the hub module.
