# Supplementary figures and images for: Left ventricular assist device bioinformatics identify possible hubgenes and regulatory networks involved in the myocardium of patients with left ventricular assist device
Source: Front Cardiovasc Med. 2022 Sep 29;9:912760. doi: 10.3389/fcvm.2022.912760 (PMC9558819; doi:10.3389/fcvm.2022.912760)

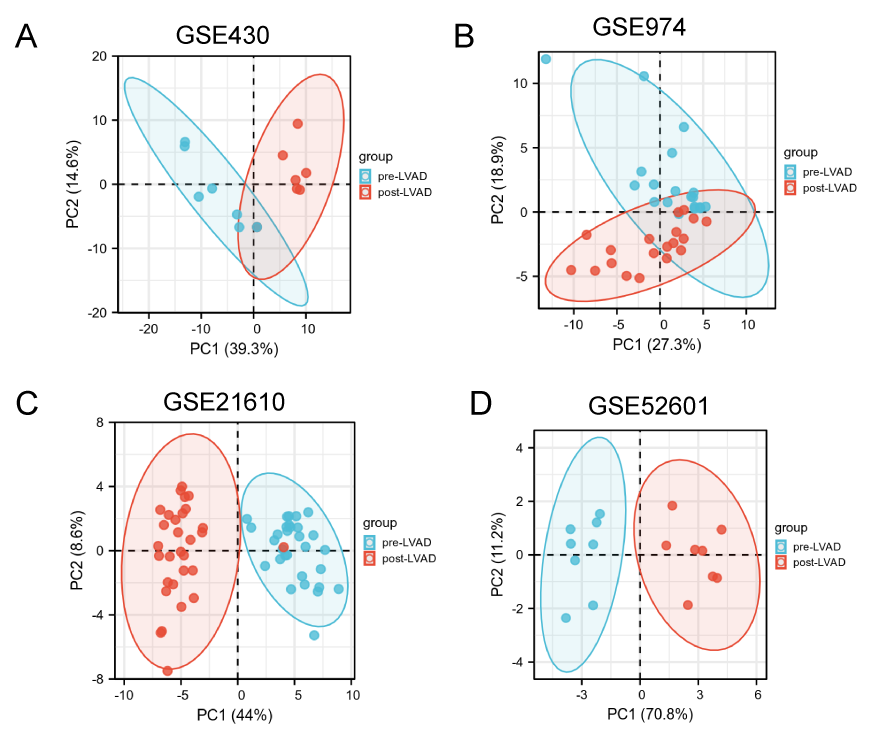

Supplement: Supplementary Figure 1 — Principal component analysis (PCA) score plots of the differentially expressed genes in each dataset, indicating that the sum of the first two principal components (PC1 and PC2) reflects the difference of more than half of the data (46.2–82%). [file Image_1.TIF]

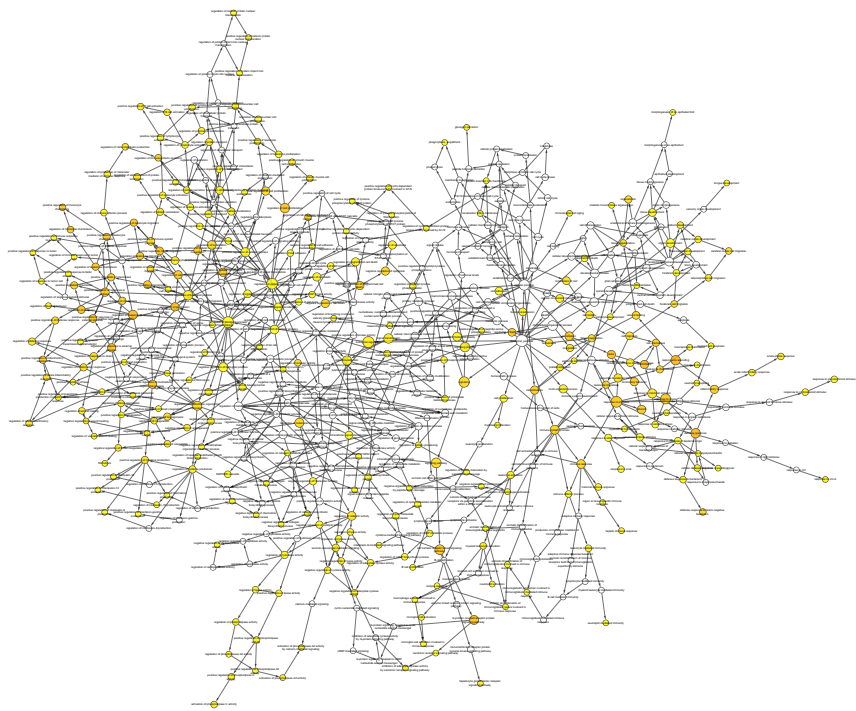

Supplement: Supplementary Figure 2 — The biological process analysis of the hubgenes in the hub module. [file Image_2.pdf]
